# MicroRNAs: Novel Players in Cancer Diagnosis and Therapies

**DOI:** 10.1155/2014/959461

**Published:** 2014-07-02

**Authors:** Aaron L. Oom, Brock A. Humphries, Chengfeng Yang

**Affiliations:** ^1^Department of Physiology, Michigan State University, East Lansing, MI 48824, USA; ^2^Cellular and Molecular Biology Graduate Program, Michigan State University, East Lansing, MI 48824, USA; ^3^Center for Integrative Toxicology, Michigan State University, East Lansing, MI 48824, USA

## Abstract

First discovered in 1993, microRNAs (miRNAs) have been one of the hottest research areas over the past two decades. Oftentimes, miRNAs levels are found to be dysregulated in cancer patients. The potential use of miRNAs in cancer therapies is an emerging and promising field, with research finding miRNAs to play a role in cancer initiation, tumor growth, and metastasis. Therefore, miRNAs could become an integral part from cancer diagnosis to treatment in future. This review aims to examine current novel research work on the potential roles of miRNAs in cancer therapies, while also discussing several current challenges and needed future research.

## 1. Introduction

MicroRNAs (miRNAs) were first discovered by Lee et al. in 1993 [1]. In the first decade after their discovery a particular focus was placed on their importance in development as first illustrated by Ambros' group. This developmental focus segued perfectly into cancer applications and has been, for the past decade, the drive for much of the research in miRNA potential use for cancer therapy.

miRNAs are small noncoding RNAs that are produced naturally by the cell. They function by sequence-specific binding of a seed sequence to the 3′ end of the untranslated region (UTR) of a target mRNA, causing the mRNA to then be degraded or to be translationally inhibited [2]. miRNAs have been thought to regulate two-thirds of the entire protein coding genome [3]. The expression of miRNAs themselves can also be regulated similarly to that of protein coding genes. Whether through genetic or epigenetic shifts, the expression levels of miRNAs are often altered in many cancers, resulting in abnormal increases or decreases [4]. These alterations have been shown to play a part in almost all facets of cancer development and progression.

Recent research has shown that tumor suppressors, such as phosphatase and tensin homolog (PTEN) [5–10] and p53 [11], can be potential targets of miRNAs. Typically downregulated in many cancers, the loss of these critical tumor suppressors can greatly increase cell proliferation and tumor progression. Furthermore, the link between some tumor suppressors and survival genes, such as the link between PTEN and the survival effector, AKT (also known as protein kinase B or PKB), may indicate therapeutic means of targeting metastasis, tumor growth, and cancer survival. And indeed, current research is examining these miRNAs that target tumor suppressors both on their own and in combination with traditional therapies, such as cisplatin [9, 10], etoposide, and ionizing radiation [11].

Additionally, certain families of miRNAs have been implicated in epithelial-to-mesenchymal transition (EMT), a critical component of cancer metastasis. EMT is typically marked by changes in morphology and cytoskeletal rearrangement. For example, one emerging family in cancer metastasis is the miR-200 family which is known for directly targeting the E-cadherin suppressors zinc finger E-box binding homeobox 1 and homeobox 2 (ZEB1 and ZEB2) [12, 13]. The loss of miR-200 family members leads to decreased E-cadherin levels. This loss of E-cadherin, an integral cell to cell adhesion protein, correlates with a dramatic increase in cells going through EMT [12, 13].

This review will examine the role that miRNAs could play in cancer therapeutics. To this end, current novel research work on the cancer therapeutic values of miRNAs will be examined.

## 2. miRNAs as Potential Markers for Cancer Diagnosis

While some of the current miRNA research work focuses on targeting novel miRNA changes and investigating the mechanism involved, other research is aimed at characterizing the miRNA expression levels for particular cancer types. It is becoming apparent that the severity and stage of cancer can be associated with miRNA levels, as seen below. This is critical, as choosing the most effective treatment option is highly dependent on precisely knowing disease aggressiveness. For example, low grade cancers do not have to be treated as aggressively as high grade cancers.

This strong drive to use miRNA screening for diagnosis serves two particular purposes: as an earlier diagnosis tool and as a more efficient means of diagnosis. Circulating miRNAs have been shown to be one of the very promising biomarkers for cancer earlier diagnosis [14]. It is believed that intracellular miRNAs are packaged into exosomes or microvesicles, which are then expelled from cells and enter the blood stream. Moreover, it has been found that circulating miRNAs are highly stable, which makes them ideal candidates serving as earlier diagnostic biomarkers for cancer [15].

For example, colorectal cancer diagnosis currently utilizes either invasive methods, such as a colonoscopy, or far less precise methods, such as fecal analysis. The often seen reluctance to comply with such measures ultimately results in much later diagnosis in patients [16]. Examining tissue and blood samples from patients of varying stages of colorectal cancer, Yong et al. showed seven miRNAs altered in both, with three of those having strong positive correlations between blood and tissue samples: miR-193a-3p, miR-23a, and miR-338-5p. Interestingly, the levels of each increased as the stage of the cancer progressed, suggesting an important role mechanistically as well as diagnostically for this trio [16].

Much like colorectal cancer, prostate cancer has long suffered from ambiguities and difficulties within diagnosis methodology. The most employed diagnosis method for prostate cancer is the Gleason scale. Tumors are differentiated based on size and histological features. There is a certain gray area when it comes to this method, making it far more difficult to choose a proper course of treatment. The use of miRNAs to categorize prostate cancer subtypes could overhaul the Gleason system and present a more accurate and reliable system. To address this, Wach et al. screened two cohorts of prostate cancer patients. They found that four particular miRNAs—miR-143, miR-145, miR-200c, and miR-375—were the most dramatically altered miRNAs in the two cohorts. Of the four, miR-143, miR-145, and miR-375 were best at distinguishing between malignant and nonmalignant tumors. Considering the three in conjunction, they were able to correctly distinguish between malignant and nonmalignant samples 77.6% of the time [17].

If a cancer type already has a standard method of subtype characterization it is possible for researchers to develop a miRNA screen around the molecular markers used for diagnosis; this was employed by Leivonen et al. with HER2 positive breast cancer lines and two patient cohorts. The group found a wide variety of miRNAs that downregulated HER2 and found a strong correlation between higher miR-342-5p levels and survival time [18]. While this type of characterization allows for a miRNA signature to be created for various cancer subtypes and thereby offering therapeutic options, it also serves the very practical purpose of diagnosis.

Of the possible benefits that can be derived from miRNA screens, the most substantial is the uniformity. We are currently able to detect miRNA levels from a basic serum sample. The power of taking a single blood sample from a patient and being able to address a wide variety of cancers from one miRNA screen is staggering.

## 3. miRNAs as Potential Cancer Therapies

Diagnosing from miRNA screens is not the only possible application; there is emerging evidence that it is possible to manipulate miRNA levels to enhance current cancer therapeutics. The miRNA screening process has allowed for the rise of more specialized research that focuses on these significantly dysregulated miRNAs and what they may be targeting. Reintroduction or inhibition of the dysregulated miRNA in conjunction with traditional cancer therapies could make for a more efficient treatment plan. This natural progression of methodology has yielded numerous miRNAs in each cancer type that can also serve as potential targets on their own in various cancers. Many miRNAs have been shown to have therapeutic potentials; in the following sections only a few selected miRNAs and target pathways are discussed to help highlight important and significant areas of the research.

### 3.1. miRNAs Targeting Receptor Tyrosine Kinases

Receptor tyrosine kinases (RTKs) are well known for their role in upregulating angiogenesis and cell proliferation in many cancers and have therefore served as critical drug targets in cancer treatment [19]. Fasanaro et al. found that miR-210 expression is significantly upregulated under hypoxic conditions. Hypoxic conditions are typical in several cancer microenvironments, such as tumor cores and bone cancers. MicroRNA-210 expression triggered the formation of capillary structures and directly targeted ephrin 3A, a ligand for a member of the RTK subfamily, ephrin receptors [20].

Comparable findings have also been found across various cancers, such as non-small cell lung cancers, breast cancer, and glioblastomas. Oneyama et al. showed that miR-99a expression significantly inhibited tumor growth and cell proliferation in lung cancers. The group showed that miR-99a was targeted by oncogenic proteins such as Ras and epidermal growth factor (EGF). Furthermore, they showed that miR-99a targets the RTK, fibroblast growth factor 3 (FGFR3) [21]. Similarly, Acunzo et al. found that miR-27a targets MET, a well-known oncogene, and epidermal growth factor receptor (EGFR) in non-small cell lung cancers [22]. Mackiewicz et al. found that miR-34a significantly reduces cell migration in breast cancer cell lines by directly targeting the RTK, AXL [23]. Likewise, Feng et al. showed that lin28 expression significantly upregulates HER2 in breast cancer and correlates with a poorer prognosis in patients when highly expressed. Furthermore, lin28 expression—which is known to target let-7a—significantly increases cell growth* in vitro* [24]. Finally, Rao et al. showed that upregulation of miR-219-5p inhibited cell growth and cell migration in glioblastoma cells via EGFR targeting [25]. Together these findings suggest an alternative method of targeting RTKs across a variety of cancers.

### 3.2. miRNAs Targeting Bcl-2 Family Members

Bcl-2 family members are either antiapoptotic or proapoptotic, with dysregulation often occurring in both types in many cancers. Kwon et al. found that miR-193a-3p directly targets the antiapoptotic Mcl-1 with stable expression of this miR inhibiting cell growth, inducing apoptosis, and creating DNA damage in the glioblastoma cell line, U-251 [26]. Zhang et al. showed that overexpression of miR-29a significantly inhibited cell proliferation and promoted apoptosis in U2OS and SAOS-2 osteosarcoma cells. This group also showed in a luciferase reporter assay that miR-29a directly inhibits Bcl-2 and Mcl-1, both antiapoptotic family members [27]. Likewise, Ji et al. found that low miR-133a levels corresponded to a poorer prognosis in osteosarcoma patients. When miR-133a was expressed* in vivo*, the group found that tumor volumes were significantly suppressed. MicroRNA-133a was found to target Bcl-xL and Mcl-1 and promoted apoptosis upon overexpression [28]. Similarly in pancreatic cancer Guo et al. showed that restoration of miR-491-5p yielded a slight increase in cell death and a slight decrease in cell proliferation by targeting Bcl-xL and TP53 in SW1990 pancreatic cancer cells [29]. Ji et al. showed that a restoration of miR-34 as well inhibited cell proliferation* in vitro* and tumor growth* in vivo* in MiaPaCa2 pancreatic cancer cells through direct Bcl-2 targeting [30].

Targeting of Bcl-2 was also found in colorectal cancer and leukemia by Zhang and Cimmino. Zhang et al. found that miR-148a significantly induced cell apoptosis by directly targeting Bcl-2 in RKO and Levo colorectal cancer cell lines [31], while Cimmino et al. showed that when miR-15 and miR-16 were reexpressed cell apoptosis was significantly upregulated in MEG-01 leukemia cells by directly targeting Bcl-2 [32]. Lastly, Lin et al. found that upregulation of miR-122 decreased relative cell numbers and significantly increased caspase-3 activity by directly targeting Bcl-w, an antiapoptotic family member, in hepatocellular carcinoma cell lines [33]. With many Bcl-2 family inhibitors still locked in clinical trials, miRNAs that target the family could prove to be another source of Bcl-2 inhibitors.

### 3.3. miRNAs Targeting the Ras Subfamily

One of the best known oncogene families is the Ras subfamily. The Ras subfamily is critically involved in cell survival, angiogenesis, and proliferation. Upregulation of Ras is found in numerous cancers and causes unchecked growth and invasiveness [34]. Thus, it serves as a valuable target in cancer treatments.

Kasinski and Slack showed that miR-34 inhibits cell proliferation and migration* in vitro* in lung cancer cells by targeting K-Ras in addition to other oncogenes. Additionally, ectopic expression of miR-34 significantly suppressed tumor growth* in vivo* [35]. Similarly, Sun et al. found that downregulation of miR-31 significantly decreased cell proliferation* in vitro* and showed a significant decrease in tumor size* in vivo* with Caco-2 and HT-29 human colorectal adenocarcinoma cells by directly targeting RAS p21 GTPase activating protein 1 (RASA1), a regulator of the RAS-MAPK pathway [36].

Shin et al. found that expression of miR-181a significantly suppressed cell proliferation and colony formation in squamous cell carcinoma cells. This group also found that miR-181a directly targets K-Ras in squamous cell carcinoma cells [37]. More specifically, Jang et al. showed that restoration of miR-636 by suppression of adenine nucleotide translocase 2 (ANT2) significantly reduces cell proliferation* in vitro* and tumor growth* in vivo* by directly targeting Ras in hepatocellular carcinoma cells [38].

Finally, Wang et al. showed that restoration of miR-451 significantly inhibits cell proliferation and colony formation and induces apoptosis in human non-small cell lung cancer cells. Additionally, expression of miR-451* in vivo* significantly reduced tumor growth. They showed that miR-451 directly targets Ras-related protein 14 (RAB14) in human non-small cell lung cancer cells [39].

Additional miRNAs having potential therapeutic values are listed in Table 1.

## 4. miRNAs as Cancer Therapy Resistance Mediators

A large amount of research has also focused on the use of miRNAs in conjunction with traditional therapies and their ability to mediate therapeutic responses. For the purpose of discussion, miRNAs that confer resistance to therapy when highly expressed will be considered resistance mediators. In the interest of space, only a few selected miRNAs are discussed in the following sections.

### 4.1. miR-21

One prominent example of a resistance mediator is miR-21. Current research suggests that one role of miR-21 in cancer is regulating DNA repair and maintenance in response to treatment, particularly nucleoside analogs. Paik and colleagues found that using a miR-21 inhibitor significantly reduced resistance to gemcitabine in Panc-1, a human pancreatic cancer cell line [78]. Likewise, Hwang and colleagues showed that inhibition of miR-21 dramatically decreased cell growth in PL45 cells, a pancreatic ductal adenocarcinoma cell line, when treated with fluorouracil (5-FU) [79]. Both 5-FU and gemcitabine are chemotherapy agents that act as nucleoside analogs, raising the possibility of a role of miR-21 in DNA repair.

To strengthen this, Wong et al. found that prolonged exposure to temozolomide, an alkylating agent, significantly upregulated miR-21 expression in D54MG glioblastoma cells. Inhibition of miR-21 drastically increased cell death when resistant D54MG cells were treated with temozolomide [81]. Additionally, Griveau et al. showed that when miR-21 was inhibited with a locked nucleic acid in U87MG glioblastoma cells, the cells were far more susceptible to radiation-induced cell death [82]. Deng et al. found that miR-21 also regulates thymidine phosphorylase, dihydropyrimidine dehydrogenase, and human DNA mismatch repair protein Msh2 (hMSH2)—proteins involved in DNA repair and synthesis—in the colon cancer cell line HT-29. When miR-21 was downregulated the cells were significantly resensitized to 5-FU treatment and irradiation [85]. Lastly, Valeri et al. showed that miR-21 was directly targeting hMSH2 and creating 5-FU resistance [86]. All together, these findings strongly suggest that miR-21 is playing an important role in cancer cell DNA repair and maintenance.

Furthermore, evidence is showing that miR-21 could be interacting with apoptosis-regulating proteins and important tumor suppressors, allowing for prolonged cell survival and unchecked growth and proliferation. Li et al. found that when miR-21 was suppressed in U373 MG glioblastoma cells, the cells were significantly resensitized to VM-26, a topoisomerase-II inhibitor. The group found that miR-21 directly targets leucine rich repeat (in FLII) interacting protein 1 (LRRFIP1), a protein with downstream products in the tumor necrosis factor receptor family [83]. Yang et al. showed that prolonged exposure to cisplatin in SGC7901 cells, a gastric cancer cell line, significantly upregulated miR-21 expression and downregulated PTEN expression. Inhibition of miR-21 resensitized the cisplatin-resistant SGC7901 cells to cisplatin, a platinum-based chemodrug that triggers DNA crosslinking [7]. Based on these findings miR-21 is acting in a wide variety of roles across a variety of cancers. The ubiquitous nature and overexpression of miR-21 in cancers make it a valuable target of great clinical significance.

### 4.2. miR-125b

The oncomiR miR-125 is of great importance in breast cancer, as there has been a growing body of evidence suggesting that it plays a crucial role in initiation, metastasis, and recurrence. Current research is slightly split on the role of miR-125b with more research suggesting that miR-125b is important in conferring therapy resistant phenotypes in a variety of cancers rather than a therapy sensitive phenotype. Zhou et al. found that inhibition of miR-125b resensitized cells to paclitaxel due to miR-125b directly targeting Bak1, a proapoptotic factor, in a suite of breast cancer cell lines [99]. Similarly, Liu et al. showed that miR-125b was significantly upregulated in taxol-resistant breast cancer cell lines. Further research has elucidated that Snail upregulates miR-125b which directly targets Bak1, thereby* reducing* chemosensitivity to gemcitabine and taxol [100]. Likewise, Wang et al. showed a negative correlation between circulating miR-125b levels in breast cancer patients and treatment response and a positive correlation between miR-125b levels and disease severity. By directly targeting the cell cycle regulator E2F3 miR-125b has been shown to increase resistance to 5-FU in a variety of breast cancer cell lines [101]. Outside of breast cancer lines, Iida et al. found that miR-125b was upregulated in doxorubicin-resistant derivatives of the Ewing sarcoma lines, WE-68 and VH-64. Once again, miR-125b was targeting Bak1 and p53, thereby decreasing chemosensitivity to doxorubicin [102].

### 4.3. miRNAs Targeting Tumor Suppressors

As previously mentioned, PTEN and p53 are critical tumor suppressors that are often dysregulated in many cancers. Emerging research suggests that a wide variety of miRNAs target PTEN and p53 allowing for cancer cells to proliferate and grow at an alarming rate. Additionally, the suppression of PTEN and p53 helps to grant a chemoresistant phenotype across several cancers. Fu et al. showed that miR-93 was significantly upregulated in CDDP resistant variations of the ovarian cancer lines OVCAR3 and SKOV3 and in primary tumor samples. Further analysis revealed that miR-93 binds to the 3′UTR of PTEN and regulates its expression [5]. The regulation of PTEN by miRNAs is also found in breast cancer. Liang et al. found that inhibition of miR-19* in vitro* and* in vivo* decreased cell viability and tumor growth, respectively. Liang et al. found miR-19 to directly target the tumor suppressor, PTEN, in MCF-7 cells [6]. Likewise, Guo et al. showed that when c-Myc is overexpressed there was a significant increase in cell viability, while inhibition of c-Myc significantly reduced cell viability in U87 and U251 glioblastoma multiforme cell lines. The group showed that the upregulation of c-Myc, a prominent oncogene, upregulates miR-26a, which then directly targets PTEN [8]. Li et al. found that miR-92b was dramatically upregulated in both non-small cell lung cancer tissue samples and cell lines. miR-92b expression was found to significantly resensitize cells to cisplatin by directly targeting PTEN [9]. Zhao et al. found that when they suppressed miR-221, a cisplatin-sensitive phenotype was restored to the osteosarcoma cell lines, SOSP-9607 and MG63. miR-221 was shown to be directly targeting PTEN in the cell lines [10]. Liu et al. found that miR-375 desensitizes the cells to etoposide, a topoisomerase inhibitor, and to ionizing radiation by directly targeting p53 in MCF-7, AGS, and A549 cell lines [11]. The expression of both of these well-known tumor suppressors is typically down in most cancers, but these findings help to elucidate a possible mechanism through which this downregulation occurs and offer clinical targets for slowing tumor progression. This ever expanding and critical overlap between oncomiRs and tumor suppressors may serve to illuminate the most effective targets for supplementing current cancer therapeutics.

## 5. miRNAs as Cancer Therapy Sensitizers

### 5.1. let-7 Interactions with Chemotherapy

Research has also uncovered several families of miRNAs that are significantly reduced in most cancers. A wealth of current research strongly suggests that the let-7 family can significantly sensitize cancer cells to therapy, reduce proliferation, reduce invasion, and reduce cell growth. Chen et al. found that the levels of let-7a in patients with acute myeloid leukemia (AML) correlated significantly with better prognosis, and upregulation of let-7a* in vitro* or* in vivo* significantly resensitized AML to cytarabine (also known as Ara-C) treatment. This group found that let-7a is regulated by CXCR4 in acute myeloid leukemia [67]. Lv et al. from the same group as Wang et al. found that lin28 regulation of let-7a also affected chemoresistance in SK-BR-3 cells. Downregulation of lin28 decreased resistance to paclitaxel and high levels of lin28 correlated with metastasis and/or relapse [68]. Bhutia et al. showed that lin28 and SET regulated processing of let-7a in pancreatic cancer cell lines. Furthermore, they found that resistance to gemcitabine correlated with a buildup of unprocessed pre-let-7 along with an increase of RRM2, a potential target of let-7a that is involved in the reduction of ribonucleotides to deoxyribonucleotides [70]. Thus, lin28 may serve as an important therapeutic target, in addition to the use of let-7 mimics.

Guo et al. showed that let-7b reconstitution restored a cisplatin-sensitive phenotype in U251 cells. Additionally, cyclin D1 is a direct target of let-7b, with knockdown of cyclin D1 yielding a phenotype similar to let-7b reconstitution [71]. Sugimura et al. found that let-7c regulates the interleukin-6/STAT3 prosurvival pathway in esophageal cancer. When let-7c was highly expressed, the IC_50_ for cisplatin was significantly decreased and the overall survival times for patients were increased [72]. Along those lines, Zhao et al. showed that higher levels of let-7c in NSCLC correlated with longer survival times in patients, with carcinoma tissues in patients typically having significantly lower levels of let-7c than normal tissues. Let-7c was shown to be directly targeting integrin *β*_3_ and MAP4K3. Restoration of let-7c expression inhibited proliferation, invasion, and migration* in vitro* [127]. Cai et al. found that cisplatin treatment downregulated let-7e expression in ovarian cancer cell lines. The group found that enhancer of zeste homolog 2 (EZH2) and cyclin D1, both thought to play a role in drug resistance, are potentially regulated by let-7e. Reexpression of let-7e* in vivo* significantly slowed tumor progression and reduced EZH2 and cyclin D1 expression [73]. The let-7 family is proving to be critical across a wide variety of cancer types, with reexpression often having significant effects, making it an excellent target for future clinical use.

### 5.2. miR-200 Family Interactions with Chemotherapies

Another emerging metastasis suppressor and therapy sensitizer is the miR-200 family. The miR-200 family, while particularly known for its role in suppressing EMT, is proving to play an additional role in mediating cancer cell response to traditional therapeutic regimens. Much of the current research on the miR-200 family has focused particularly on miR-200a, -200b, and -200c, with far less research looking at miR-141 and miR-429. Soubani et al. showed that the curcumin analog, difluorinated curcumin (CDF), is able to upregulate miR-200a, -200b, and -200c in pancreatic cancer lines. Additionally, it was shown that the expression of CDF upregulated the critical tumor suppressor PTEN [128]. Furthering this, Ali et al. found that CDF-mediated upregulation of miR-200b and miR-200c along with CDF-mediated downregulation of miR-21 elevated PTEN levels and suppressed NF-*κ*B DNA binding activity. The modulations of these microRNAs significantly resensitized pancreatic cancer cells to gemcitabine [80].

Kopp et al. found that reconstitution of miR-200c in breast cancer significantly sensitized cells to doxorubicin. Furthermore, miR-200c expression was found to be targeting Bmi1 and TrkB, a potential oncogene and a survival factor, respectively [116]. Cochrane et al. showed that reexpression of miR-200c in ovarian cancer—where expression is typically reduced—significantly resensitized cells to paclitaxel [117]. On the other hand, Prislei et al. showed that miR-200c levels can have varying effects on ovarian cancer. The group showed that TUBB3, a potential target of miR-200c, has both a 200c binding site and a HuR binding site. Depending on the location of HuR, patients with high miR-200c levels had either a better (nuclear HuR) or worse (cytoplasmic HuR) prognosis, with regard to survival time and progression free survival, due to differential interactions of miR-200c with TUBB3 [129]. Lastly, Hur et al. found that restoration of miR-200c to miR-200c deficient colorectal cancer lines yielded a significant increase in cell proliferation. However, there was a marked decrease in cell invasion and migration, suggesting a critical role for miR-200c in suppressing an EMT phenotype. Through luciferase reporter assay the group found that miR-200c targets ZEB1, protein C-ets-1 (ETS1), and vascular endothelial growth factor receptor 1 (FLT1) [130].

### 5.3. Various miRNA Interactions with Irradiation

While a vast amount of current research focuses on miRNAs in combination with chemotherapy, a small body of studies examines irradiation treatments in conjunction with miRNA treatment. The alteration of miRNAs involved in DNA damage repair due to radiation would allow for cancer cells to resist radiation treatment [131–133]. To this extent, Huang et al. focused on RAD51 and its paralog RAD51D, two proteins involved in homologous recombination mediated double strand break repair. The group found that miR-107, which directly targeted RAD51 and RAD51D, and miR-103 when upregulated in the human osteosarcoma cell line U20S sufficiently resensitized cells to irradiation and several chemotherapeutics [96].

Wang et al. showed that overexpression of lin28, a cancer stem cell marker, in SK-BR-3 breast cancer cells, downregulated let-7a. Restoration of let-7a expression significantly resensitized cells to radiation treatment [69]. Wang et al. found that miR-23b expression was down in radiation resistant lines of pancreatic cancer cells. When reexpressed, cells were far more sensitive to radiation treatment both* in vitro* and* in vivo*. The group found that miR-23b targets ATG12, thereby regulating autophagy [88].

Zhang et al. showed that miR-29c is frequently downregulated in nasopharynx cancers. Restoration of typical miR-29c levels significantly resensitized cells to irradiation and cisplatin* in vitro* and significantly reduced tumor growths* in vivo*. It was shown that miR-29c suppresses both Mcl-1 and Bcl-2 [89]. Yang et al. found that miR-145 is typically downregulated in glioblastoma multiforme patients. Reexpression of miR-145 created a temozolomide and irradiation sensitive phenotype* in vitro* and* in vivo*, with an impressively significant reduction in tumor growth. MiR-145 was found to directly target both Oct4 and Sox2 [108]. Liang et al. showed that miR-302 replacement therapy significantly resensitized breast cancer cells to irradiation* in vivo* and* in vitro* by directly targeting AKT1 and RAD52 [120].

A more comprehensive look at miRNA interactions with traditional therapies can be found in Table 2.

## 6. miRNA Delivery Systems

One of the issues to be confronted with miRNAs in treatment is the system of delivery. A variety of means from nanotechnology to lipids to viruses have been explored, each with its own advantages and setbacks.

### 6.1. Nanotechnology-Based miRNA Delivery Systems

Much of the current research on miRNA delivery is trending towards the use of nanotechnologies. Ando et al. found that the use of dicetyl phosphate-tetraethylenepentamine-based polycation liposomes was significantly more effective than N-[1-(2,3-dioleoyloxy)propyl]-N,N,N-trimethylammonium methylsulfate-based liposomes (DOTAP) for delivering miR-92a to human umbilical vein endothelial cells (HUVECs) and releasing it into the cytoplasm [134]. Biray et al. showed that the use of polyethylene glycol-polyethylenimine nanocomplexes was approximately 80% more effective than control reagents in delivering miR-150 into chronic myeloid leukemia cells [135]. Likewise, Yang et al. found that the use of cationic polyurethane-short branch polyethylenimine for miR-145 significantly reduced glioblastoma multiforme tumor sizes* in vivo*. Impressively, the addition of irradiation and temozolomide nearly removed all traces of tumors [108]. Lastly, Cao et al. showed that protamine sulfate-nanodiamond hybrid nanoparticle delivery of miR-203 significantly reduced cell migration and proliferation in the esophageal cancer cell line, Ec-109 [136]. The emergence of nanotechnology as a delivery mechanism, particularly within the last year, is significant for clinical development of miRNA therapies.

### 6.2. Lipid-Based miRNA Delivery Systems

In the past, cationic lipid and polymer based delivery systems have suffered from cytotoxic side effects* in vivo*, mainly due to their cationic nature. Many of these systems contain common organic structures, creating biochemical consequences in the cell [137]. However, recent advances in the field have yielded promising results. Griveau et al. found that lipid nanocapsule-locked nucleic acid complexes targeting miR-21 in glioblastoma cells significantly resensitized the cells to irradiation at 48 hours after transfection as compared to N-TER Nanoparticle siRNA Transfection System, a current transfection reagent [82]. Similarly, Shi et al. found that solid lipid nanoparticles were effective in delivering anti-miR-21 oligonucleotides to lung cancer cells* in vitro*. This group saw a significant decrease in migration and invasion along with a significant increase in apoptosis of these cancer cells [138]. Moreover, Trang et al. showed that a neutral lipid emulsion containing either miR-34a or let-7b significantly reduced lung tumor growth* in vivo* [139]. Piao et al. showed that lipid-based nanoparticles were effective in delivering pre-miR-107 to neck and squamous cancer cells* in vivo*. Analysis of the tumors revealed a significant reduction in tumor growth and an increase in survival [140]. Current advances may make lipid-based delivery systems viable methods of delivery within the next few years.

### 6.3. Virus-Based miRNA Delivery Systems

Viral delivery systems, while far more efficient than other mechanisms, frequently elicit immunogenic responses, hindering their overall effectiveness. Because of this significant limitation, viral delivery systems have been primarily limited to* in vitro* work, with retroviruses playing an important role in RNAi research. Current research on the practicality of viral delivery systems for miRNAs is extremely limited and as such is only mentioned for the sake of acknowledgement.

## 7. Challenges and Perspectives

While miRNAs have a wealth of potential, the field has several challenges that it still needs to address. There are limitations for using circulating miRNAs as potential biomarkers for cancer earlier diagnosis. One of the limitations is the reported inconsistence of circulating miRNA alterations in a particular type of cancer. For example, the levels of a good number of (>30) miRNAs have been found to be significantly changed (increased or decreased) in gastric cancer patients' blood, serum, or plasma samples [141]. Nevertheless, the observed alterations of circulating miRNAs are mostly sporadic with little consensus among different studies. Few circulating miRNAs were found to be similarly and significantly altered in three or more than three studies.

As is usually a problem with most pharmaceutics, miRNAs in therapy would require an effective delivery system. Much of the current research for this is focusing on various nanotechnologies in hopes of reducing the toxicity seen in some current delivery mechanisms. The selected delivery systems must meet several criteria with no major issues of safety and efficient use. Much of the current research on the safety of RNA interference* in vivo* revolves around shRNAs, but there may still be some applicability in the results. Grimm et al. found that overexpression of over 30 different shRNAs* in vivo* caused liver toxicity and ultimately death in several mice. The group found that miRNAs and shRNAs were competing for cellular processing equipment leading to a buildup of premature miRNAs and shRNAs [142].

Many research groups reintroduce miRNAs through the use of pre-miRNAs. This influx of unprocessed miRNAs could flood the miRNA processing system and lead to toxic, and potentially lethal, side effects. However, Liu et al. showed that while lentiviral delivery of miR-30 to melanomas had comparable effects on cancer progression* in vivo* similar to shRNAs, miR-30 had little incidence of inflammation [143]. So it is possible that miRNAs could attenuate some of the side effects of shRNAs, but this is a gap in knowledge that will need filling as the field moves closer to clinical applications. However, this hurdle of investigating safety and miRNA delivery* in vivo* is just beginning to be met and is the next major challenge to the field on its way to full clinical application.

The field of miRNA use in cancer therapy is most likely heading in a direction that is more oriented towards* in vivo* investigation and translational research. However, presently, there are some cancers that are far more represented in the literature than others. The current literature is more geared towards cancers such as glioblastomas, breast, ovarian, non-small cell lung, and pancreatic than it is towards cancers like kidney and leukemia. It is the hope of the authors that these gaps in research will soon be filled in, bringing all cancers to a comparable level of understanding. However, the future of the field is a bright one and clinical applications will hopefully come to fruition within the next decade.

## Figures and Tables

**Table 1:** miRNAs explored as potential cancer therapeutic agents.

miR	Cancer type	Targets
let-7a	Breast [24], endometrial [40]	HER2 and aurora-B
miR-15 and miR-16	Leukemia [32]	Bcl-2
miR-21	Glioblastoma, breast [41]	E-cadherin-ZEB1/2 pathway
miR-21 and miR-181b	Glioma [42]	FOS
miR-22 and miR-200b	Gastric [43]	Wnt-1 pathway
miR-26a	Liver, prostate, skin [44], bladder [45]	Lin28B, Zcchc11, and HMGA1
miR-27a	Lung [22], breast [46]	MET, EGFR, and PI3K-AKT pathway
miR-27b	Colorectal [47]	VEGFC
miR-29a	Osteosarcoma [27]	Bcl-2, Mcl-1
miR-31	Colorectal [36]	RASA1
miR-34	Breast (34a) [23], pancreatic [30], lung [35], lung (34a, c) [48]	AXL, Bcl-2, K-Ras, and PDGFR-*α*/*β*
miR-99a	Lung [21]	mTOR/FGFR3
miR-106b-5p	Glioma [49]	RBL1, RBL2, and CASP8
miR-122	Liver [33]	Bcl-w
miR-130b	Pancreatic [50]	STAT3
miR-133a	Osteosarcoma [28]	Bcl-xL and Mcl-1
miR-138	Glioblastoma [51]	EZH2-CDK4/6-pRb-E2F1 pathway
miR-145	Ovarian [52]	p70S6K1 and MUC1
miR-148a	Colorectal [31], liver [53]	Bcl-2 and Met/Snail pathway
miR-150	Lung [54]	p53
miR-155	Breast [46, 55, 56]	VHL, TP53INP1, and PI3K-AKT pathway
miR-181	Squamous cell (181a) [37], breast [57]	K-Ras and ataxia telangiectasia mutated (ATM)
miR-185 and miR-342	Prostate [58]	SREBP pathway
miR-193a-3p	Glioblastoma [26]	Mcl-1
miR-205	Oral [59]	Axin 2
miR-210	Hypoxic conditions [20]	Ephrin-A3
miR-219-5p	Glioblastoma [25]	EGFR
miR-221	Pancreatic [60], breast [61]	PTEN, p27kip1, p57kip2, and PUMA
miR-301a	Breast [62]	PTEN
miR-449a and -449b	Retinoblastoma [63]	E2F transcription factors
miR-451	Lung [39]	RAB14
miR-491-5p	Pancreatic [29]	TP53 and Bcl-xL
miR-494	Glioma [64]	p190B RhoGAP
miR-497	Neuroblastoma [65]	WEE1
miR-636	Liver [38]	Ras
miR-708	Bladder [66]	Caspase-2

**Table 2:** miRNAs in combination with traditional cancer therapies.

miR	Cancer type (effect of expression on treatment)	Treatments investigated
let-7a	Leukemia [67] (sensitive), breast [68, 69] (sensitive), pancreatic [70] (sensitive)	Ara-C, irradiation, paclitaxel, gemcitabine
let-7b	Glioblastoma [71] (sensitive)	Cisplatin
let-7c	Esophageal [72] (sensitive)	Cisplatin
let-7e	Ovarian [73] (sensitive)	Cisplatin
miR-9	Glioblastoma [74] (resistant)	Temozolomide
miR-10b	Colon [75] (resistant)	Fluorouracil
miR-15b	Tongue [76] (sensitive)	Gemcitabine
miR-17-5p	Pancreatic [77] (resistant)	Gemcitabine
miR-19a	Breast [6] (resistant)	Taxol, mitoxantrone, etoposide
miR-21	Gastric [7] (resistant), pancreatic [78–80] (resistant), glioblastoma [81–84] (resistant), colorectal [85, 86] (resistant), adenocarcinoma [87] (resistant)	Gemcitabine, fluorouracil, temozolomide, irradiation, cisplatin
miR-23b	Pancreatic [88] (sensitive)	Irradiation
miR-29c	Nasopharynx [89] (sensitive)	Cisplatin, irradiation
miR-30c	Breast [90] (sensitive)	Paclitaxel, doxorubicin
miR-30d, miR-181a and miR-199a-5p	Colorectal, prostate, and leukemia [91] (sensitive)	Trichostatin A
miR-31	Ovarian [92] (sensitive)	Paclitaxel
miR-34a	Breast [93] (sensitive), prostate [94] (sensitive), gastric [95] (miR-34c-5p, sensitive)	Adriamycin, camptothecin, paclitaxel
miR-92b	Lung [9] (resistant)	Cisplatin
miR-93	Ovarian [5] (resistant)	Cisplatin
miR-103 and miR-107	Osteosarcoma, cervical, lung [96] (sensitive)	AZD2281, cisplatin, etoposide, camptothecin, irradiation
miR-106a	Ovarian [97, 98] (sensitive or resistant)	Cisplatin, paclitaxel
miR-125b	Breast [99–101] (resistant), Ewing sarcoma [102] (sensitive)	Paclitaxel, fluorouracil, doxorubicin, gemcitabine
miR-128-2	Lung [103] (resistant)	Cisplatin, doxorubicin, fluorouracil
miR-130a	Liver [104] (resistant)	Cisplatin
miR-140	Osteosarcoma and colon [105] (resistant)	Fluorouracil
miR-141	Ovarian [106] (resistant)	Cisplatin
miR-143 and miR-145	Colon [107] (sensitive)	Fluorouracil, irinotecan, oxaliplatin
miR-145	Glioblastoma [108] (sensitive), cervical [109] (sensitive)	Irradiation, temozolomide, mitomycin
miR-152 and miR-185	Ovarian [110] (sensitive)	Cisplatin
miR-181b	Pancreatic [111, 112] (sensitive or resistant), glioblastoma [113] (sensitive)	Gemcitabine, temozolomide
miR-182	Ovarian [114] (resistant)	Paclitaxel, cisplatin
miR-199a-5p	Liver [115] (sensitive)	Cisplatin
miR-200b	Pancreatic [80] (sensitive), tongue [75] (sensitive)	Gemcitabine, cisplatin
miR-200c	Pancreatic [80] (sensitive), breast [116] (sensitive), ovarian [117] (sensitive)	Gemcitabine, paclitaxel, doxorubicin
miR-223	Liver [118] (sensitive)	Doxorubicin, paclitaxel
miR-298	Breast [119] (sensitive)	Doxorubicin
miR-302	Breast [120] (sensitive)	Irradiation
miR-320	Prostate [121] (sensitive), pancreatic [122] (miR-320c, resistant)	Cisplatin, carboplatin, paclitaxel, gemcitabine
miR-375	Gastric [11] (resistant), cervical [123] (resistant)	Irradiation, etoposide, paclitaxel
miR-591	Ovarian [97] (sensitive)	Paclitaxel
miR-650	Lung [124] (resistant)	Docetaxel
miR-663	Breast [125] (resistant)	Adriamycin, chlorophosphamide, docetaxel
miR-708	Ewing sarcoma [126] (sensitive)	Etoposide, doxorubicin
